# A Novel Diterpene Glycoside with Nine Glucose Units from *Stevia rebaudiana* Bertoni

**DOI:** 10.3390/biom7010010

**Published:** 2017-01-31

**Authors:** Indra Prakash, Gil Ma, Cynthia Bunders, Romila D. Charan, Catherine Ramirez, Krishna P. Devkota, Tara M. Snyder

**Affiliations:** 1 The Coca-Cola Company, One Coca-Cola Plaza, Atlanta, GA 30313, USA; gilma@coca-cola.com (G.M.); cynbun@gmail.com (C.B.); 2 AMRI-Albany, Analytical Development, Albany, NY 12212, USA; Romila.Charan@amriglobal.com (R.D.C.); Catherine.Ramirez@amriglobal.com (C.R.); Krishna.Devkota@amriglobal.com (K.P.D.); Tara.Snyder@amriglobal.com (T.M.S.)

**Keywords:** Diterpene glycosides, nine sugar steviol glycoside, structure elucidation, NMR, *Stevia rebaudiana* Bertoni, 1→6 glycoside linkage

## Abstract

Following our interest in new diterpene glycosides with better taste profiles than that of Rebaudioside M, we have recently isolated and characterized Rebaudioside IX—a novel steviol glycoside—from a commercially-supplied extract of *Stevia rebaudiana* Bertoni. This molecule contains a hexasaccharide group attached at C-13 of the central diterpene core, and contains three additional glucose units when compared with Rebaudioside M. Here we report the complete structure elucidation—based on extensive Nuclear Magnetic Resonance (NMR) analysis (^1^H, ^13^C, Correlation Spectroscopy (COSY), Heteronuclear Single Quantum Coherence-Distortionless Enhancement Polarization Transfer (HSQC-DEPT), Heteronuclear Multiple Bond Correlation (HMBC), 1D Total Correlation Spectroscopy (TOCSY), Nuclear Overhauser Effect Spectroscopy (NOESY)) and mass spectral data—of this novel diterpene glycoside with nine sugar moieties and containing a relatively rare 1→6 α-linked glycoside. A steviol glycoside bearing nine glucose units is unprecedented in the literature, and could have an impact on the natural sweetener catalog.

## 1. Introduction

Humans are born with a predisposition to like sweet tastes. Sweet taste has always been associated with pleasure, and consumers enjoy eating or drinking sweet-tasting items. Recently, health-conscious consumers have placed an increasing priority on reducing their caloric intake without compromising sweetness. This has led the food industry to search for novel natural high-potency sweeteners. In the past ten years, we have looked to the leaves of *Stevia rebaudiana* Bertoni for sweet-tasting steviol glycosides [1,2,3]. This family of natural products which possesses a diterpene core has been used to sweeten food products for hundreds of years in South America [4]. However, by the early 21st century, the chemical structures of only a limited number of steviol glycosides had been characterized. These included stevioside, Rebaudioside A–F, dulcoside A, and steviolbioside [5]. In recent years, a greater number of minor steviol glycosides have been identified that are present in trace quantities in dried extracts of *S. rebaudiana* leaves [6,7,8,9,10,11]. However, the taste profile and physico-chemical properties of most isolated and purified steviol glycosides have not been reported.

Recently, we have focused our efforts on finding new steviol glycosides with a superior taste profile and similar structure to Rebaudioside M. Rebaudioside M—one of the minor components in *S. rebaudiana* leaf extract—shows an improved sweet taste profile, reduced perception of bitterness, reduced astringency, and reduced lingering bitterness compared with that of other known steviol glycosides, including Rebaudioside A [12,13]. Our most recent work uncovered a unique Rebaudioside M derivative with a hydroxyl group at the C-15 position of the diterpene core [14]. In addition, we have developed biotransformation reactions to produce Rebaudioside M and Rebaudioside M2 [15]. 

**Figure 1:**
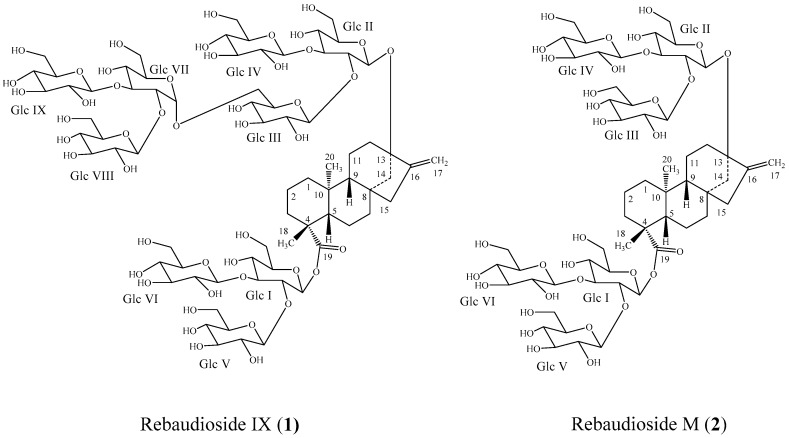
Structures of Rebaudioside IX (**1**) and Rebaudioside M (**2**).

In our continued search to find potentially significant sweet diterpene glycosides that are present only in minor abundance in *S. rebaudiana*, we have isolated a novel diterpene glycoside from commercial leaf extracts provided by PureCircle (Negeri Sembilan, Malaysia). The novel compound, Rebaudioside IX, has nine glucose units, in which the (2-*O*-β-d-glucopyranosyl-3-*O*-β-d-glucopyranosyl)-d-glucopyranosyl moiety is attached to Glc III of Rebaudioside M as a 1→6 sugar linkage in an α-configuration. To the best of our knowledge, a steviol glycoside with nine glucose units is unprecedented in the scientific literature, and the 1→6 α-linkage present in Rebaudioside IX is relatively rare in the steviol glycoside family. This paper describes the isolation and structure elucidation of the novel nine-sugar diterpene glycoside (**1**) (Figure 1) on the basis of extensive spectroscopic studies. 

## 2. Results and Discussion

Compound **1** was isolated as a white powder. Accurate mass measurement using high resolution mass spectrometry (HRMS) provided an exact mass at *m*/*z* 1775.7295 in the negative electrospray ionization-time-of-flight (ESI-TOF) mass spectrum corresponding to a molecular formula of C_74_H_120_O_48_ (calculated for C_74_H_119_O_48_: 1775.6871). HRMS and Nuclear Magnetic Resonance (NMR) data indicated that (**1**) had an additional glucotriosyl unit when compared with Rebaudioside M (**2**).

**Figure 2:**
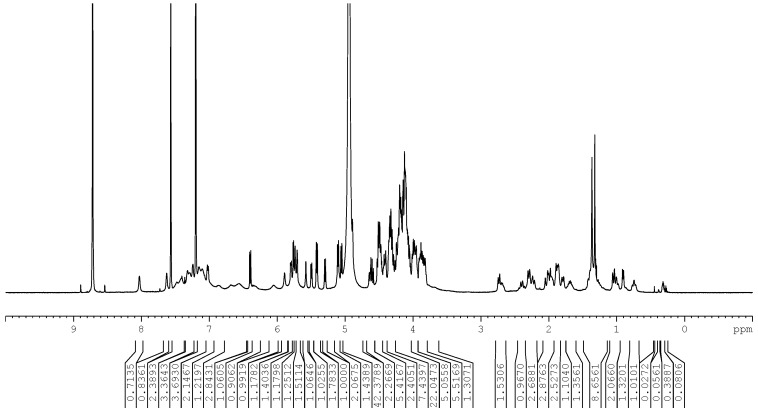
^1^H NMR spectrum of Rebaudioside IX (**1**) (500 MHz, 300 K) in pyridine-*d*_5_. ppm: parts per million.

A series of NMR experiments including ^1^H NMR, ^13^C NMR, ^1^H-^1^H correlation spectroscopy (COSY), ^1^H-^13^C Heteronuclear Single Quantum Coherence-Distortionless Enhancement Polarization Transfer (HSQC-DEPT), ^1^H-^13^C Heteronuclear Multiple Bond Correlation (HMBC), ^1^H-^1^H Nuclear Overhauser Effect Spectroscopy (NOESY), and 1D Total Correlation Spectroscopy (TOCSY) were performed to allow the elucidation of the structure of (**1**) (see Supplementary Materials for representative 1D and 2D NMR spectra). The 1D and 2D NMR data indicated that the central core of the glycoside was a diterpene. The ^1^H NMR spectrum (Figure 2) and the HSQC-DEPT data of (**1**) indicated the presence of two methyl singlets at δ 1.33 and 1.37, two proton singlets corresponding to an exocyclic double bond at δ_H_ 4.89 and 5.71, nine methylene and two methine protons between δ_H_ 0.75–2.74 characteristic for the *ent-*kaurene diterpene central core, and similar to data reported for Rebaudioside M and other steviol glycosides [4,7]. The presence of an *ent-*kaurene diterpenoid aglycone central core was further confirmed by ^1^H-^1^H COSY correlations of H-1/H-2; H-2/H-3; H-5/H-6; H-6/H-7; H-9/H-11; H-11/H-12 and ^1^H-^13^C HMBC correlations of H-3/C-2, C-4, C-18; H-5/C-4, C-6, C-10, C-19; H-9/C-8, C-10, C-11, C-12, C-14, C-15, C-20; H-12/C-13; H-14/C-15, C-16; H-15/C-13, C-16, H-17/C-13, C15; H-18/C-3, C-4, C-5, C-19; H-20/C-1, C-5, C-9, C-10. The complete ^1^H and ^13^C assignments of the central diterpene core of (**1**) are provided in Table 1. 

The relative stereochemistry in the central diterpene core was assigned based on Nuclear Overhauser Effect (NOE) correlations. In the NOESY spectrum of (**1**), NOE correlations between H-14/H-20 indicated that H-14 and H-20 were on the same face of the rings. Similarly, NOE correlations between H-9/H-5 as well as H-5/H-18, but an absence of NOE correlations between H-9/H-14 indicated that H-5, H-9, and H-18 were on the opposite face of the rings to that of H-14 and H-20, as presented in Figure 1. These data thus indicated that the relative stereochemistry in the central diterpene core of (**1**) was retained during glycosylation, and is the same as that for Rebaudioside M (Figure 1).

The ^1^H NMR and the ^1^H-^13^C HSQC-DEPT data for (**1**) showed signals for nine anomeric protons at δ_H_ 6.40 (δ_C_ 95.3), δ_H_ 5.80 (δ_C_ 104.6), 5.76 (δ_C_ 100.6), 5.48 (δ_C_ 104.8), 5.42 (δ_C_ 96.6), 5.40 (δ_C_ 104.3), 5.29 (δ_C_ 104.6), 5.10 (δ_C_ 106.5), and 5.06 (δ_C_ 105.4), indicating the presence of nine glucose units. This was supported by fragment ions in the negative Electrospray Ionization Tandem Mass Spectrometry (ESI MS/MS) spectrum of (**1**), which showed the loss of one hexose unit at *m/z* 1613.6594, followed by the loss of two hexose units at *m/z* 1289.5540 and the sequential loss of six hexose moieties at *m/z* 1127.4983, 965.4431, 803.3970, 641.3356, 479.2852, and 317.2128. 

**Figure 3:**
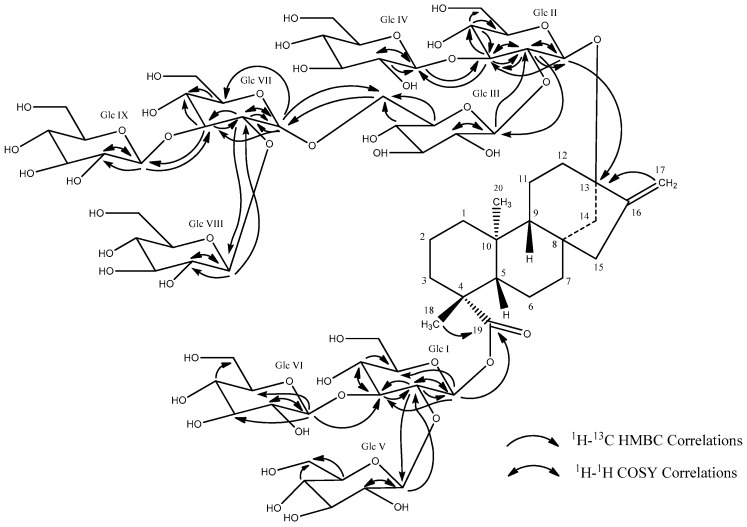
Summary of key Heteronuclear Multiple Bond Correlation (HMBC) and Correlation Spectroscopy (COSY) correlations used to assign the C-13 and C-19 glycoside region of compound (**1**).

The complete assembly of the glycoside structure was determined on the basis of the COSY, HSQC-DEPT, and HMBC correlations, as well as 1D TOCSY data. The 1D TOCSY experiments were used extensively to assign protons of the nine glucose units, while the HSQC-DEPT and HMBC data allowed the carbon assignments and established the connectivity of the glucose units. Thus, long-range ^1^H-^13^C correlations observed in the HMBC experiment from the anomeric proton at δ_H_ 6.40 (δ_C_ 95.3) to a carbonyl carbon at δ_C_ 177.3 (C-19) allowed its assignment as the anomeric proton of Glc I (Figure 2). The ^1^H coupling sequence from Glc I anomeric proton (δ_H_ 6.40) to H-2 (δ_H_ 4.51) through H-3 (δ_H_ 5.11), H-4 (δ_H_ 4.20), H-5 (δ_H_ 4.14), and the oxymethylene protons, H-6 (δ_H_ 4.20 and 4.32), was established using a combination of COSY and 1D TOCSY data. The carbons C-2 (δ_C_ 77.2), C-3 (δ_C_ 89.0), C-4 (δ_C_ 70.5 or 70.6 or 70.7), C-5 (δ_C_ 78.9), and C-6 (δ_C_ 62.2) were then assigned based on HSQC-DEPT data and confirmed by HMBC correlations of H-1/C-3, C-5; H-2/C-1, C-3; H-3/C-2; H-4/C-5. The higher frequency ^1^H and ^13^C chemical shifts of the C-2 and C-3 positions suggested the possible placement of glycosyl units at these positions, which was confirmed by HMBC correlations. Thus, long-range ^1^H-^13^C correlations observed in the HMBC experiment from the anomeric protons at δ_H_ 5.80 (Glc V, H-1) to the carbon at δ_C_ 77.2 (Glc I, C-2) and δ_H_ 5.29 (Glc VI, H-1) to the carbon at δ_C_ 89.0 (Glc I, C-3) established the 2,3-*O*-branched-d-glucodiosyl substituent in Glc I to be the same as that in Rebaudioside M. NMR data did not indicate any further sugar linkages to Glc I, Glc V, and Glc VI. The large coupling constants for the anomeric protons of Glc I (δ_H_ 6.40, d, *J*^3^ = 8.2 Hz), Glc V (δ_H_ 5.80, d, *J*^3^ = 7.4 Hz), and Glc VI (δ_H_ 5.29, d, *J*^3^ = 7.9 Hz) indicated their β-configuration. The complete ^1^H and ^13^C assignments of Glc V and Glc VI was based on extensive analysis of 1D and 2D NMR data, and are provided in Table 2, while the key COSY and HMBC correlations are provided in Figure 3.

Further analysis of the 1D and 2D NMR data allowed the assignment of the remaining six sugars in (**1**). The HMBC correlation from the anomeric proton at δ_H_ 5.42 (δ_C_ 96.6 via HSQC-DEPT) to a quaternary carbon at δ_C_ 88.0 (C-13) allowed its assignment as the anomeric proton of Glc II (Figure 2). The ^1^H coupling sequence from the Glc II anomeric proton (δ_H_ 5.42) to H-2 (δ_H_ 4.19) through H-3 (δ_H_ 4.93), H-4 (δ_H_ 4.09), H-5 (δ_H_ 3.88), and the oxymethylene protons, H-6 (δ_H_ 4.19 or 4.22 and 4.33), was established using a combination of COSY and 1D TOCSY data. The carbons C-2 (δ_C_ 81.2), C-3 (δ_C_ 88.6), C-4 (δ_C_ 70.5 or 70.6 or 70.7), C-5 (δ_C_ 78.0–78.5), and C-6 (δ_C_ 62.2–63.4) were then assigned based on HSQC-DEPT data and confirmed by HMBC correlations of H-1/C-3; H-2/C-1, C-3; H-3/C-2, C-4; H-4/C-3, C-5, C-6. As in Glc I, the higher-frequency ^1^H and ^13^C chemical shifts of the C-2 and C-3 positions suggested glycosyl substituents at these positions in Glc II, and this was confirmed by HMBC correlations. Thus, in Glc II, long-range ^1^H-^13^C correlations from the anomeric proton at δ_H_ 5.48 (Glc III, H-1) to the carbon at δ_C_ 81.2 (Glc II, C-2) and the anomeric proton at δ_H_ 5.40 (Glc IV, H-1) to the carbon at δ_C_ 88.6 (Glc II, C-3) in the HMBC experiment established the 2,3-*O*-branched-d-glucodiosyl substituent in Glc II to be the same as that present in Rebaudioside M. The large 3-bond coupling constants for the anomeric protons of Glc II (δ_H_ 5.42, d, *J*^3^ = 8.0 Hz), Glc III (δ_H_ 5.48, d, *J*^3^ = 7.4 Hz), and Glc IV (δ_H_ 5.40, d, *J*^3^ = 8.0 Hz) indicated their β-configuration. The key COSY and HMBC correlations are provided in Figure 2, and the complete ^1^H and ^13^C NMR assignments of the sugars are provided in Table 3. While the NMR data did not indicate any further sugar linkages to Glc II or Glc IV, the relatively higher frequency of C-6 (δ_c_ 72.1) in Glc III and HMBC correlations from Glc III H-6 (δ_H_ 4.44 and 4.50) to a carbon at δ_C_ 100.6 suggested a sugar linkage at this position, and this was subsequently confirmed by HMBC correlations. 

One of the three remaining sugars had an anomeric proton with a smaller coupling (δ_H_ 5.76, d, *J*^3^ = 3.5 Hz) suggesting an α-linked sugar in the structure, while the other two had large couplings (δ_H_ 5.10, d, *J*^3^ = 7.7 Hz and δ_H_ 5.06, d, *J*^3^ = 7.8 Hz) suggesting a β-configuration. The anomeric proton at δ_H_ 5.76 showed a long-range ^1^H-^13^C correlation to the carbon at δ_C_ 72.1 (Glc III, C-6), thus establishing a 1→6 α-linkage between Glc VII and Glc III (Figure 2). The ^1^H coupling sequence from the Glc VII anomeric proton (δ_H_ 5.76) to H-2 (δ_H_ 4.13) through H-3 (δ_H_ 4.60), H-4 (δ_H_ 4.16), H-5 (δ_H_ 4.41), and the oxymethylene protons, H-6 (δ_H_ 4.36 and ~4.5), was established using a combination of COSY and 1D TOCSY data. The Glc VII carbons C-2 (δ_C_ 81.4), C-3 (δ_C_ 84.7), C-4 (δ_C_ 70.5 or 70.6 or 70.7), C-5 (δ_C_ 74.0), and C-6 (δ_C_ 62.2–63.4) were then assigned based on HSQC-DEPT data and confirmed by HMBC correlations of H-1/C-2, C-3, C-5; H-2/C-1, C-3; H-3/C-2, C-4; H-5/C-4. The placement of the two remaining glucose moieties with anomeric protons at δ_H_ 5.10 (δ_C_ 106.5) and δ_H_ 5.06 (δ_C_ 105.4) was established by the HMBC correlations from the anomeric protons at δ_H_ 5.10 (Glc VIII, H-1) to Glc VII C-2 (δ_C_ 81.4) and δ_H_ 5.06 (Glc IX, H-1) to Glc VII C-3 (δ_C_ 84.7), thus indicating the attachment of these two sugars at C-2 and C-3, respectively, of Glc VII. Reciprocal HMBC correlations from Glc VII, H-2 to δ_C_ 106.5 (Glc VIII, C-1), and H-3 to δ_C_ 105.4 (Glc IX, C-1) further confirmed this assignment. In Glc VIII, COSY correlations between the anomeric proton (δ_H_ 5.10) to H-2 (δ_H_ 4.06) through H-3 (δ_H_ 4.17) established the assignment of Glc VIII H-2 and H-3, while H-4 (δ_H_ 4.10 or 4.16), H-5 (δ_H_ 3.81 or 3.85), and the oxymethylene protons, H-6 (δ_H_ ~4.2 to ~4.5) were assigned based on 1D TOCSY data. The carbons at C-2 (δ_C_ 75.7), C-3 (δ_C_ 78.7–79.0), C-4 (δ_C_ 72.1 or 74.0), C-5 (δ_C_ 78.7–79.0), and C-6 (δ_C_ 62.2–63.4) were assigned based on HSQC-DEPT and HMBC data. Glc IX was similarly assigned. COSY correlations between Glc IX anomeric proton (δ_H_ 5.06) and the proton at δ_H_ 3.98 established the assignment of Glc IX, H-2 resonance Glc IX H-3 (δ_H_ 4.10), H-4 (δ_H_ 4.10 or 4.16), H-5 (δ_H_ 3.81 or 3.85), and the oxymethylene protons, H-6 (δ_H_ ~4.2 to ~4.5), were assigned on the basis of 1D TOCSY data. Carbons C-2 (δ_C_ 76.1), C-3 (δ_C_ 78.7–79.0), C-4 (δ_C_ 72.1 or 74.0), C-5 (δ_C_ 78.7–79.0), and C-6 (δ_C_ 62.2–63.4) were assigned based on HSQC-DEPT and HMBC data. Some ^1^H and ^13^C chemical shifts could not be unequivocally assigned due to peak overlap, and for these, either an alternate chemical shift value or a specific range of values is provided. 

Thus, extensive analysis of the 1D and 2D NMR data established the connectivity and complete assignments of all nine sugars in this novel glycoside. The key COSY and HMBC correlations of **1** are provided in Figure 3, and the complete ^1^H and ^13^C assignments are provided in Table 1, Table 2 and Table 3. 

## 3. Experimental Section

### 3.1. General Experimental Procedures for Rebaudioside IX *(**1**)*

#### 3.1.1. Isolation and Purification

Isolation of (**1**) by Preparative High-Performance Liquid Chromatography (HPLC): The purification of 18.3 g of stevia extract was performed in three chromatographic steps (see Supplementary Materials for representative HPLC traces from these steps). All processes used a Waters Symmetry Shield RP18 (30 × 150 mm, 7 µm, p/n WAT248000; Water Corporation, Milford, MA, USA) column at ambient temperature; flow rate at 40 mL/min; detection by UV (210 nm). All gradient steps were linear. All mobile phases were premixed by volume, and contained 0.1% acetic acid (HOAc) (*v*/*v*). The primary process used the following method: Mobile Phase A: 20% acetonitrile (MeCN) in water; Mobile Phase B: 30% MeCN in water; Gradient: 0–10 min (100A:0B), 25–30 min (0A:100B), 32 min (100A:0B); Injection load: 3 g of stevia extract dissolved in 30 mL of dimethylsulfoxide (DMSO), then diluted with 90 mL of water with 0.1% HOAc. The fraction of interest (containing (**1**) based on chromatographic and MS profiles, see below for methods) was concentrated from the eluent via rotary evaporation (R-114 Rotary Evaporator; Buchi Corporation, New Castle, DE, USA). The concentrated aqueous solution was loaded “as is” in the next purification step. The second chromatographic step used the following method: Mobile Phase A: 15% MeCN in water; Mobile Phase B: 30% MeCN in water; Gradient: 0–5 min (100A:0B), 25–30 min (0A:100B), 32 min (100A:0B); Injection load: 75 mL of the aqueous sample concentrate. Fractions of interest were pooled, concentrated in vacuo, and lyophilized (FTS System benchtop lyophilizer; Labconco Corporation, Kansas City, MO, USA), yielding 62 mg of a solid powder. The third chromatographic step used an isocratic mobile phase consisting of 18% MeCN in water; Injection load: 62 mg of sample dissolved in 10 mL of water. The isolated compound was partially concentrated from the eluent via rotary evaporation, then further concentrated on a solid phase extraction (SPE) cartridge, followed by rotary evaporation and lyophilization. The purity of the final product was 90.3% as confirmed by Liquid Chromatography-Charged Aerosol Detection (LC-CAD). Approximately 4.5 mg of (**1**) was available for spectroscopic and spectrometric analyses.

Fraction analysis: analysis of preparative purification fractions were performed using the following method. Synergi Hydro-RP column (4.6 × 250 mm, 4 µm, p/n 00B-4375-A0; Phenomenex, Torrance, CA, USA); Column Temp: 55 °C; Mobile Phase A: 0.00284% NH_4_OAc and 0.0116% HOAc in water; Mobile Phase B: MeCN; Flow Rate: 1.0 mL/min; Injection volume: 10 µL. Detection was by UV (210 nm) and CAD. Primary fractions were analyzed using the following method: Gradient: 0–8.5 min (75A:25B), 10 min (71A:29B), 16.5 min (70A:30B), 18.5–23 min (66A:34B), 23.5–27.0 min (30A:70B), 27.5 min (75A:25B). Fractions from secondary and tertiary purification steps were analyzed using the following method: Gradient: 0–5 min (90A:10B), 20 min (70A:30B), 25–27 min (50A:50B), 28–30 min (90A:10B). Gradient segments were linear. The peak for (**1**) was observed at a retention time (tR) of approximately 19.7 min.

Liquid Chromatography-Mass Spectrometry(LC-MS) Analyses: LC-MS analysis of enhanced fractions was carried out on a AB Sciex API 150EX mass spectrometer (Applied Biosystems SCIEX, Waltham, MA, USA) using Turbo Spray with a mass window of 500–2000 Da using the following method. Synergi Hydro-RP (Phenomenex); Column Temp: Ambient; Mobile Phase A: 15% MeCN in water; Mobile Phase B:30% MeCN in water; Gradient: 0–5 min (100A:0B), 5–25 min (0A:100B), 25–30 min (0A:100B), 32 min (100A:0B), Flow Rate: 1.0 mL/min; Injection volume: 10 µL.

#### 3.1.2. Mass Spectrometry

The ElectroSpray Ionization-Time-Off-Flight (ESI-TOF) mass spectra and MS/MS data were generated with a Waters Q-Tof Premier mass spectrometer (Waters Coporation) equipped with an electrospray ionization source. The sample was diluted with H_2_O-MeCN (1:1) by 50-fold and introduced via infusion using the onboard syringe pump and analyzed by negative ESI.

#### 3.1.3. Nuclear Magnetic Resonance Spectroscopy

The samples of Rebaudioside IX (**1**) (~5 mg in 700 µL of pyridine-*d*_5_ or ~2 mg in 180 µL of pyridine-*d*_5_) were prepared, and NMR data were acquired on Bruker Avance 500 MHz (Bruker BioSpin, Billerica, Massachusetts, USA) instrument with a 5 mm broad band probe and 2.5 mm inverse detection probe. The ^1^H NMR and ^13^C NMR spectra were referenced to the pyridine signal at δ_H_ 8.72 and δ_C_ 150.35 ppm, respectively. All NMR data were acquired at 300 K (see Supplementary Materials for examples of representative 1D and 2D NMR spectra not presented in the main text).

### 3.2. Material Sources

The material used for the isolation of compound (**1**) was a commercial *Stevia rebaudiana* Bertoni extract, VSPC-2973-60, received from PureCircle, Malaysia.

## 4. Conclusions 

Based on extensive spectroscopic studies, the structure of the novel diterpene glycoside (**1**) with nine glucose units was established as 13-[(2-*O*-(6-*O*-α-d-glucopyranosyl-(2-*O*-β-d-glucopyranosyl-3-*O*-β-d-glucopyranosyl)-β-d-glucopyranosyl-3-*O*-β-d-glucopyranosyl)-β-d-glucopyranosyl)oxy] *ent*-kaur-16-en-19-oic acid-[(2-*O*-β-d-glucopyranosyl-3-*O*-β-d-glucopyranosyl-β-d-glucopyranosyl) ester]. Evaluation of the NMR data led to the conclusion that this compound has an additional glucotriosyl moiety—(2-*O*-β-d-glucopyranosyl-3-*O*-β-d-glucopyranosyl)-β-d-glucopyranosyl—attached at Glc III of Rebaudioside M as a 1→6 sugar linkage in an α-configuration. To the best of our knowledge, this is the first report of a steviol glycoside with nine sugars. In addition to this novel feature, the α 1→6 sugar linkage present in Rebaudioside IX is also rare for this class of compounds. Rebaudioside IX is an important finding, and expands our understanding of the diversity of the diterpene glycosides in the leaves of *S. rebaudiana* Bertoni. The finding demonstrates that new structurally unique Rebaudiosides can still be isolated and identified from *S. rebaudiana*, and that the approach exemplified here offers opportunities to find new sweeteners for the health-conscious consumer.

## Figures and Tables

**Table 1:** ^1^H and ^13^C Nuclear Magnetic Resonance (NMR) (500 and 125 MHz, 300 K) assignments of the aglycone of compound (**1**) in pyridine-*d*_5_.

Atom No.	^13^C NMR	^1^H NMR
1	40.7	0.75 m, 1.86 m
2	20.1	1.36 m, 2.29 m
3	38.9	1.01 m, 2.30 m
4	44.7	-
5	57.9	1.05 d (13.1)
6	23.9	2.23 m, 2.39 m
7	43.0	1.40 m, 1.80 m
8	41.6	-
9	54.8	0.91 d (7.9)
10	40.2	-
11	20.6	1.68 m, 1.88 m
12	39.0	1.95 m, 2.69 m
13	88.0	-
14	43.9	1.99 m, 2.74 d (10.6)
15	46.9	1.87 m, 2.02 m
16	153.7	-
17	105.4	4.89 bs, 5.71 bs
18	28.7	1.33 s
19	177.3	-
20	17.1	1.37 s

**Table 2:** ^1^H and ^13^C NMR (500 and 125 MHz, 300 K) assignments of the C-19 glycosides of compound (**1**) in pyridine-*d*_5_.

Sugar	Atom No.	^13^C NMR	^1^H NMR
Glc I	1	95.3	6.40 d (8.2)
	2	77.2	4.51 m
	3	89.0	5.11 m
	4	70.5 or 70.6 or 70.7	4.20 m
	5	78.9	4.14 m
	6	62.2	4.20 m, 4.32 m
Glc V	1	104.6	5.80 d (7.4)
	2	75.8	4.19 m
	3	78.0–78.5 ^¥^	4.20 m
	4	71.5 or 71.6 or 72.1	4.11 m
	5	78.0–78.5 ^¥^	3.90 m
	6	64.4	4.33 m, 4.63 m
Glc VI	1	104.6	5.29 d (7.9)
	2	75.9	3.95 m
	3	78.0–78.5 ^¥^	4.35 m
	4	71.5 or 71.6	4.09 m
	5	78.0–78.5 ^¥^	3.86 m
	6	62.2–63.4 ^¥^	4.10 m or 4.36 m, 4.30 m

^¥^ Chemical shifts could not be unequivocally assigned due to very close chemical shifts or overlapping resonances.

**Table 3:** ^1^H and ^13^C NMR (500 and 125 MHz, 300K) assignments of the C-13 glycosides of compound **1** in pyridine-*d*_5_.

Sugar	Atom No.	^13^C NMR	^1^H NMR
Glc II	1	96.6	5.42 d (8.0)
	2	81.2	4.19 m
	3	88.6	4.93 m ^#^
	4	70.5 or 70.6 or 70.7	4.09 m
	5	78.0–78.5 ^¥^	3.88 m
	6	62.2–63.4 ^¥^	4.19 m or 4.22 m, 4.33 m
Glc III	1	104.8	5.48 d (7.4)
	2	75.9 or 76.0	4.11 m
	3	78.7–79.0 ^¥^	4.13 m
	4	74.3	3.87 m
	5	76.5	3.97 m
	6	72.1	4.44 m, 4.50 m
Glc IV	1	104.3	5.40 d (8.0)
	2	75.9 or 76.0	3.97 m
	3	78.7–79.0 ^¥^	4.47 m
	4	71.5 or 71.6	4.13 m or 4.15 m
	5	78.0–78.5 ^¥^	3.97 m
	6	62.2–63.4 ^¥^	4.19 m or 4.22 m, 4.32 m
Glc VII	1	100.6	5.76 d (3.5)
	2	81.4	4.13 m
	3	84.7	4.60 m
	4	70.5 or 70.6 or 70.7	4.16 m
	5	74.0	4.41 m
	6	62.2–63.4 ^¥^	4.36 m, ~4.5 m
Glc VIII	1	106.5	5.10 d (7.7)
	2	75.7	4.06 m
	3	78.7–79.0 ^¥^	4.17 m
	4	72.1 or 74.0	4.10 m or 4.16 m
	5	78.7–79.0 ^¥^	3.81 m or 3.85 m
	6	62.2–63.4 ^¥^	~4.2 m to ~4.5 m
Glc IX	1	105.4	5.06 d (7.8)
	2	76.1	3.98 m
	3	78.7–79.0 ^¥^	4.13 m
	4	72.1 or 74.0	4.10 m or 4.16 m
	5	78.7–79.0 ^¥^	3.81 m or 3.85 m
	6	62.2–63.4 ^¥^	~4.2 m to ~4.5 m

^¥^ Chemical shifts could not be unequivocally assigned due to very close chemical shifts or overlapping resonances. ^#^ Proton resonance obscured by the water resonance, assignment based on 1D Total Correlation Spectroscopy (TOCSY) and 2D NMR data.

## Supplementary Materials

The following are available online at www.mdpi.com/2218-273X/7/1/10/S1, Figure S1: Preparative HPLC trace from primary chromatographic process, Figure S2: Preparative HPLC trace from second chromatographic process, Figure S3: Preparative HPLC trace from third chromatographic process, Figure S4: ^1^H Nuclear Magnetic Resonance (NMR) spectra, full scale and expansions of Rebaudioside IX (**1**), Figure S5: ^13^C NMR spectrum of Rebaudioside IX (**1**), Figure S6: ^1^H-^1^H Correlation Spectroscopy (COSY) spectrum of Rebaudioside IX (**1**), Figure S7: ^1^H-^13^C Heteronuclear Single Quantum Coherence-Distortionless Enhancement Polarization Transfer (HSQC-DEPT) spectrum of Rebaudioside IX (**1**), Figure S8: ^1^H-^13^C Heteronuclear Multiple Bond Correlation (HMBC) spectra, full scale and expansion of Rebaudioside IX (**1**), Figure S9: ^1^H-^1^H Nuclear Overhauser Effect Spectroscopy (NOESY) spectrum of Rebaudioside IX (**1**).
