# Supplementary material for: A Novel Diterpene Glycoside with Nine Glucose Units from Stevia rebaudiana Bertoni
Source: Biomolecules. 2017 Jan 31;7(1):10. doi: 10.3390/biom7010010 (PMC5372722; doi:10.3390/biom7010010)
Supplement: Supplementary file 1 [file biomolecules-07-00010-s001.pdf]

Supplementary Materials: A Novel Diterpene Glycoside with Nine Glucose Units from *Stevia rebaudiana* Bertoni

Indra Prakash, Gil Ma, Cynthia Bunders, Romila D. Charan, Catherine Ramirez, Krishna P. Devkota and Tara M. Snyder

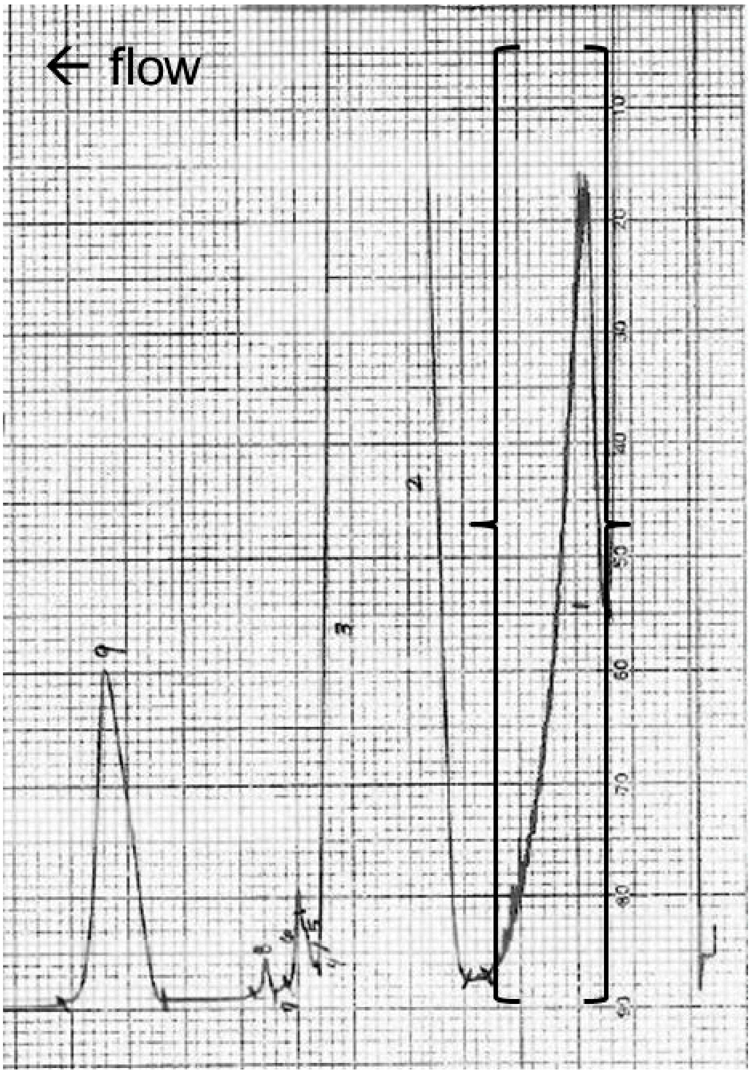

Figure S1. Preparative High-Performance Liquid Chromatography (HPLC) trace from primary chromatographic process. Target fraction (Fraction 1) is identified in brackets.

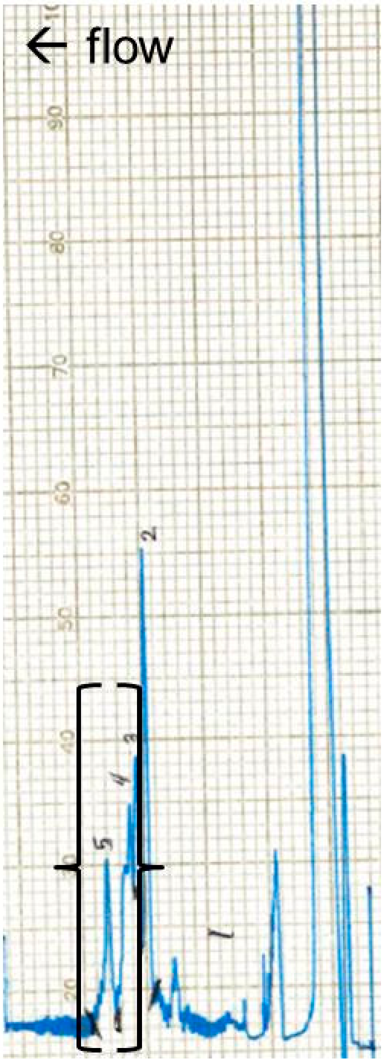

Figure S2. Preparative HPLC trace from second chromatographic process. Target fractions (Fractions 4 and 5) are identified in brackets.

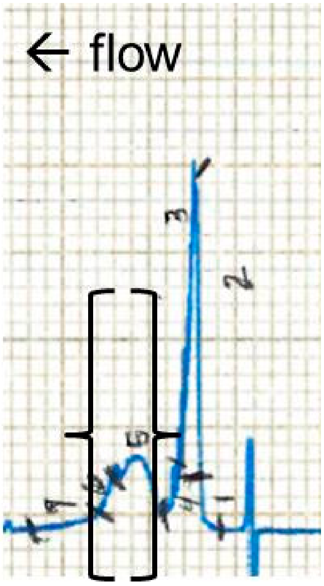

Figure S3. Preparative HPLC trace from third chromatographic process. Target fractions (Fractions 5 and 6) are identified in brackets.

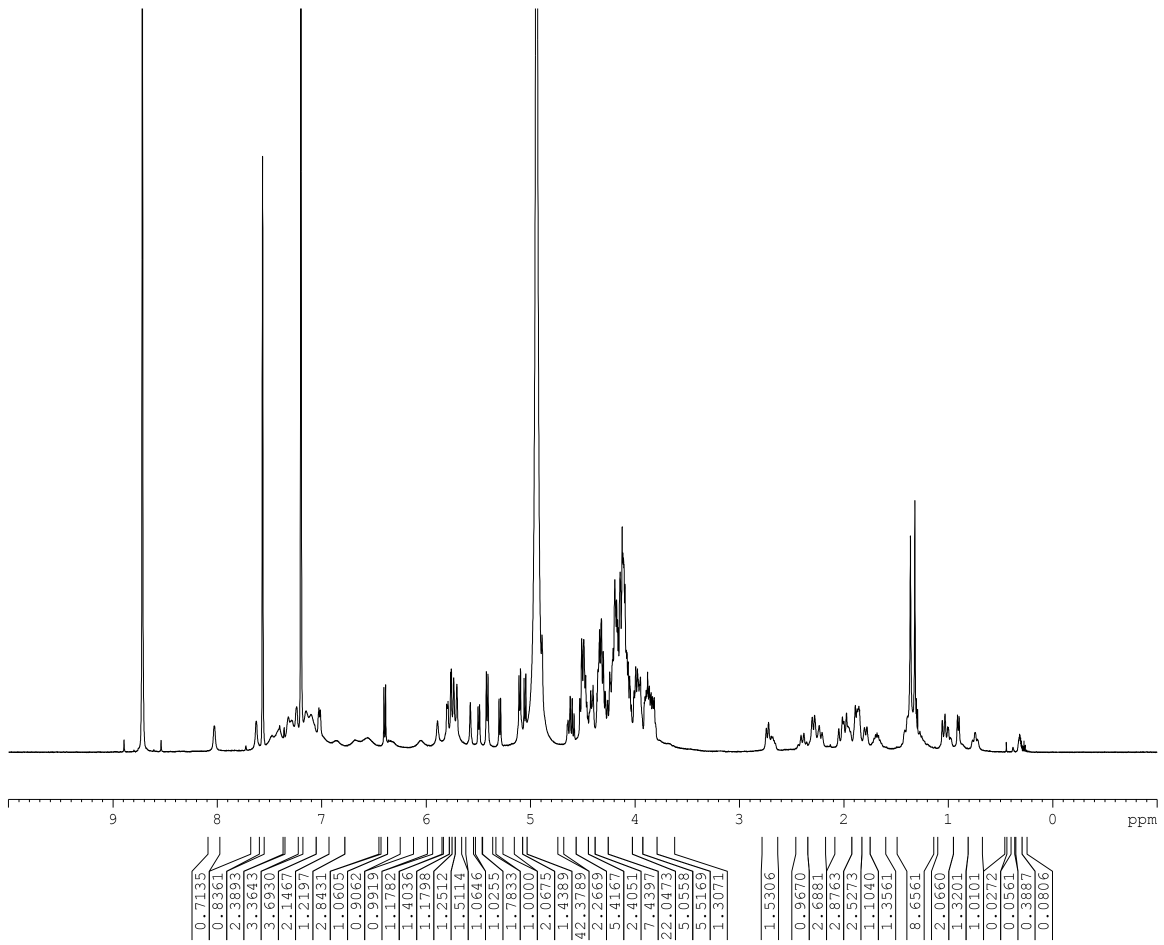

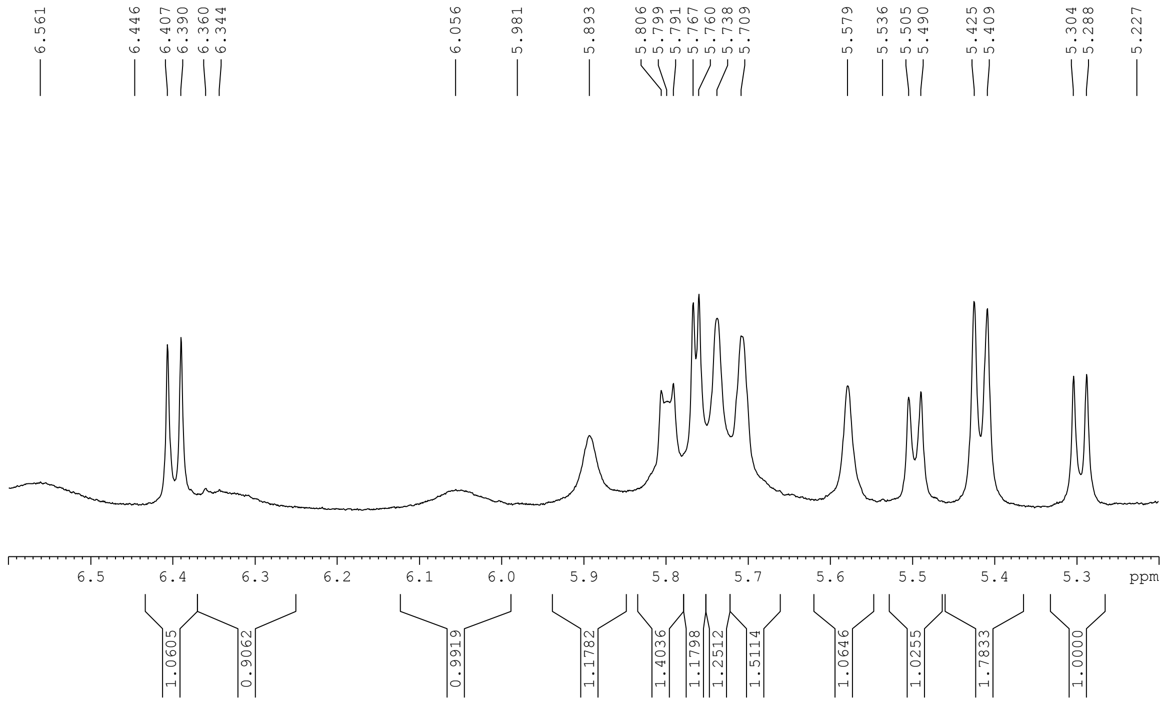

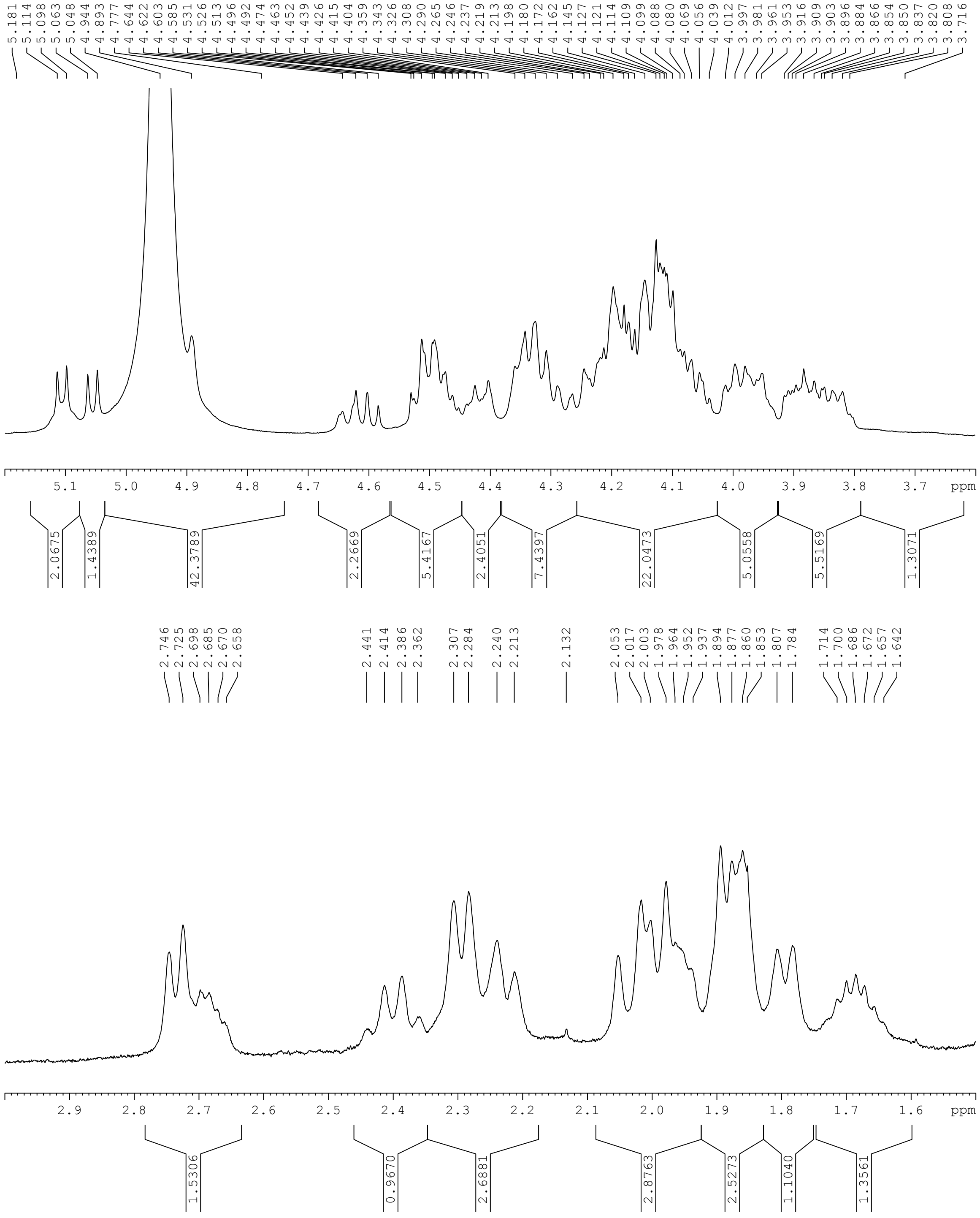

Figure S4. [1]H Nuclear Magnetic Resonance (NMR) spectra, full scale and expansions of Rebaudioside IX (**1**).

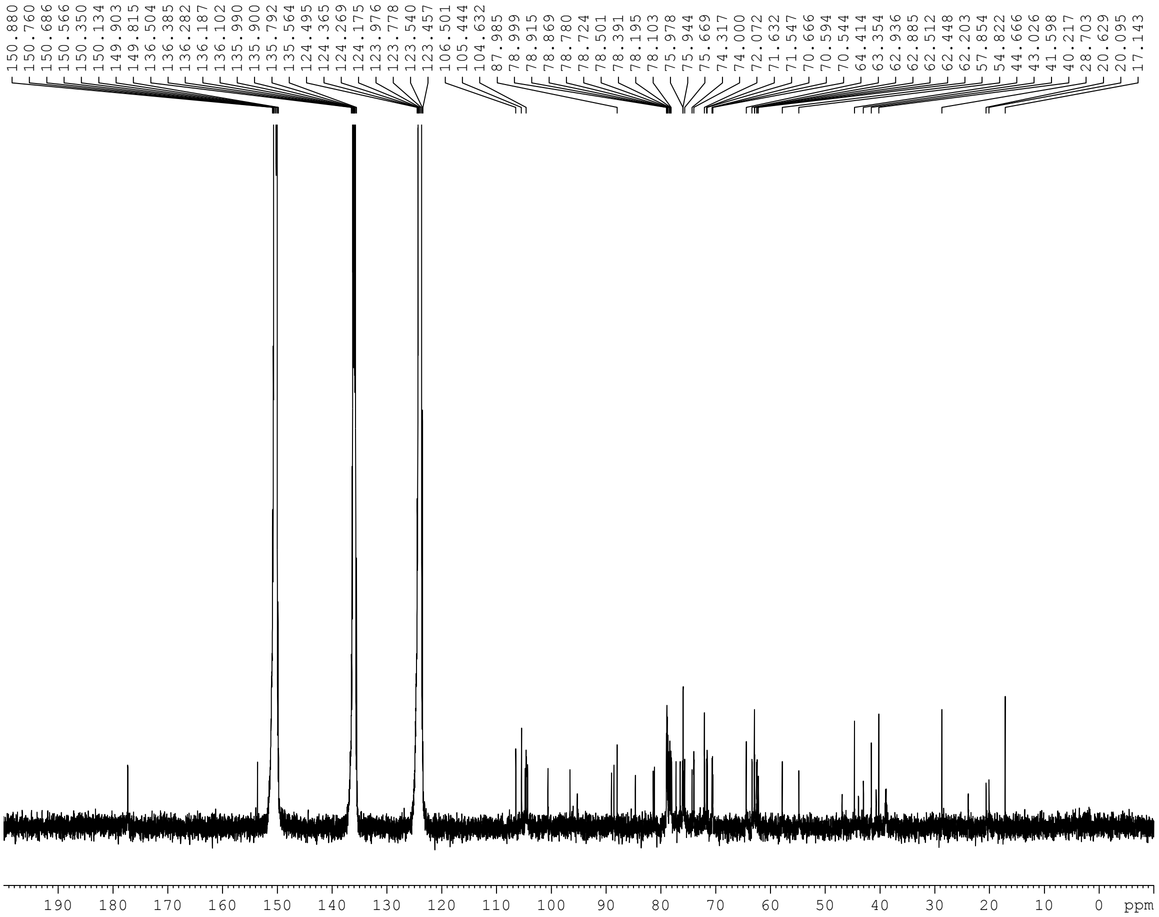

Figure S5. ^{13}C NMR spectrum of Rebaudioside IX (**1**).

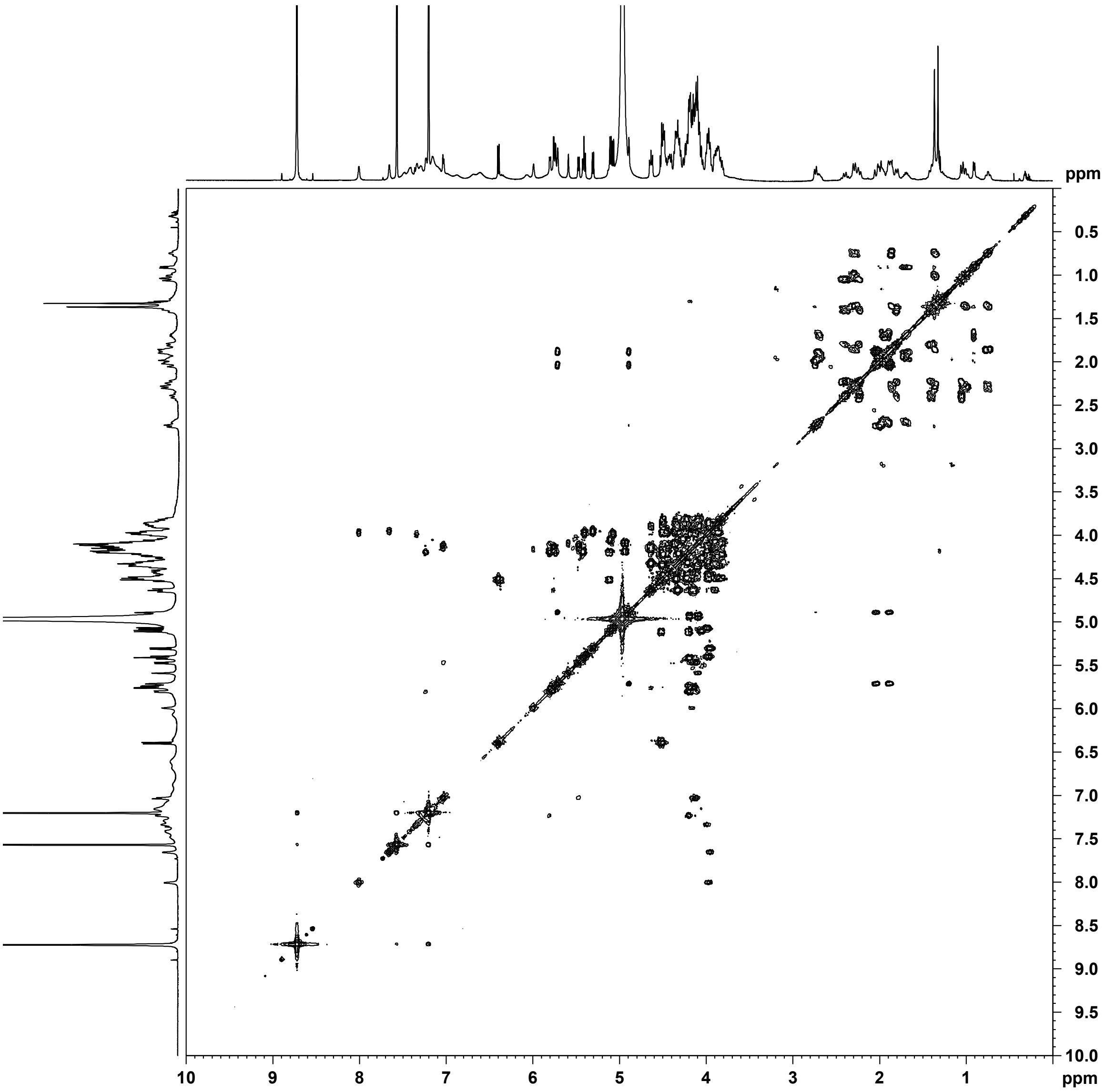

Figure S6. ^{1}H-[1]H Correlation Spectroscopy (COSY) spectrum of Rebaudioside IX (**1**).

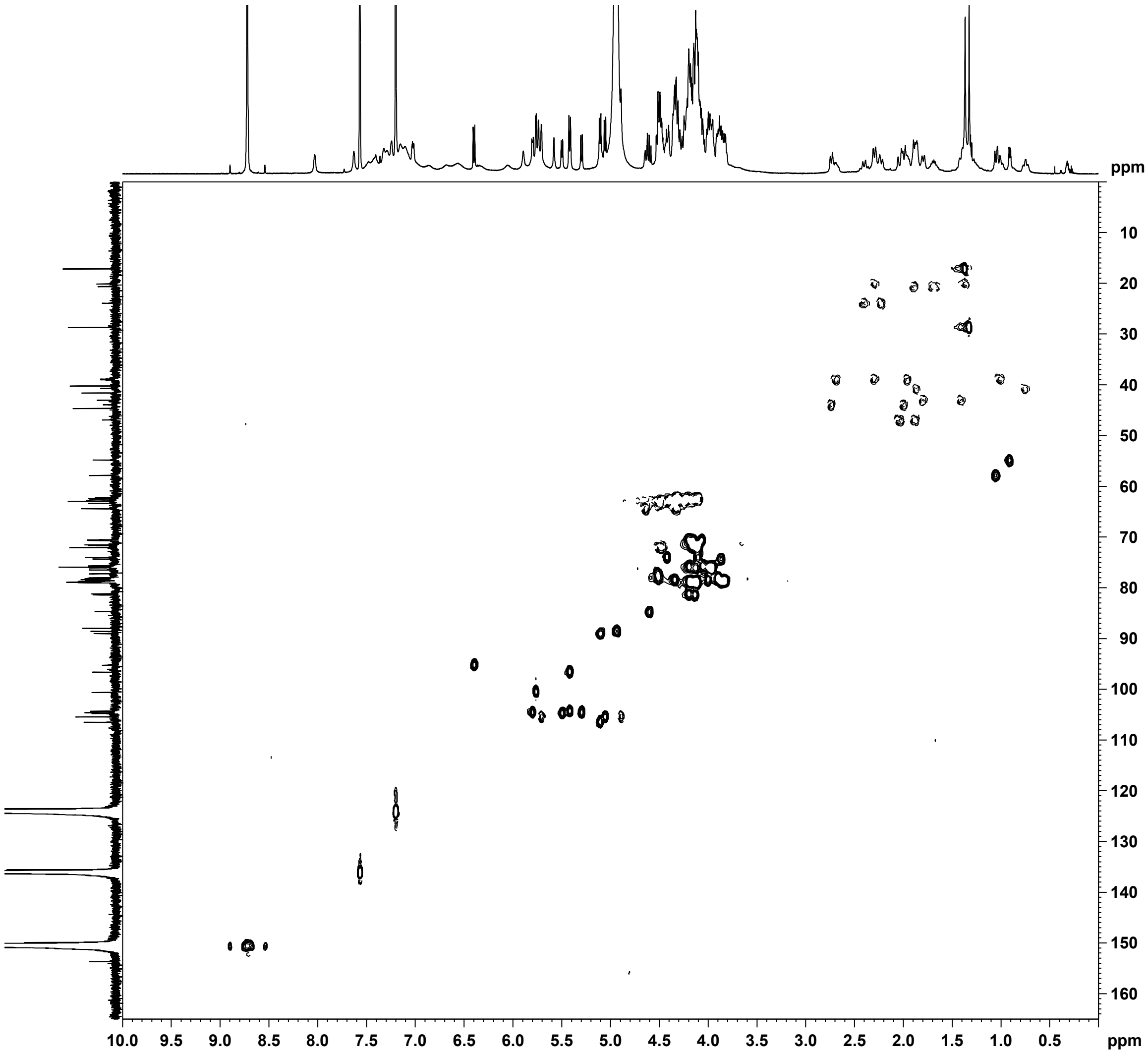

Figure S7. ^{1}H-^{13}C Heteronuclear Single Quantum Coherence-Distortionless Enhancement Polarization Transfer (HSQC-DEPT) spectrum of Rebaudioside IX (**1**).

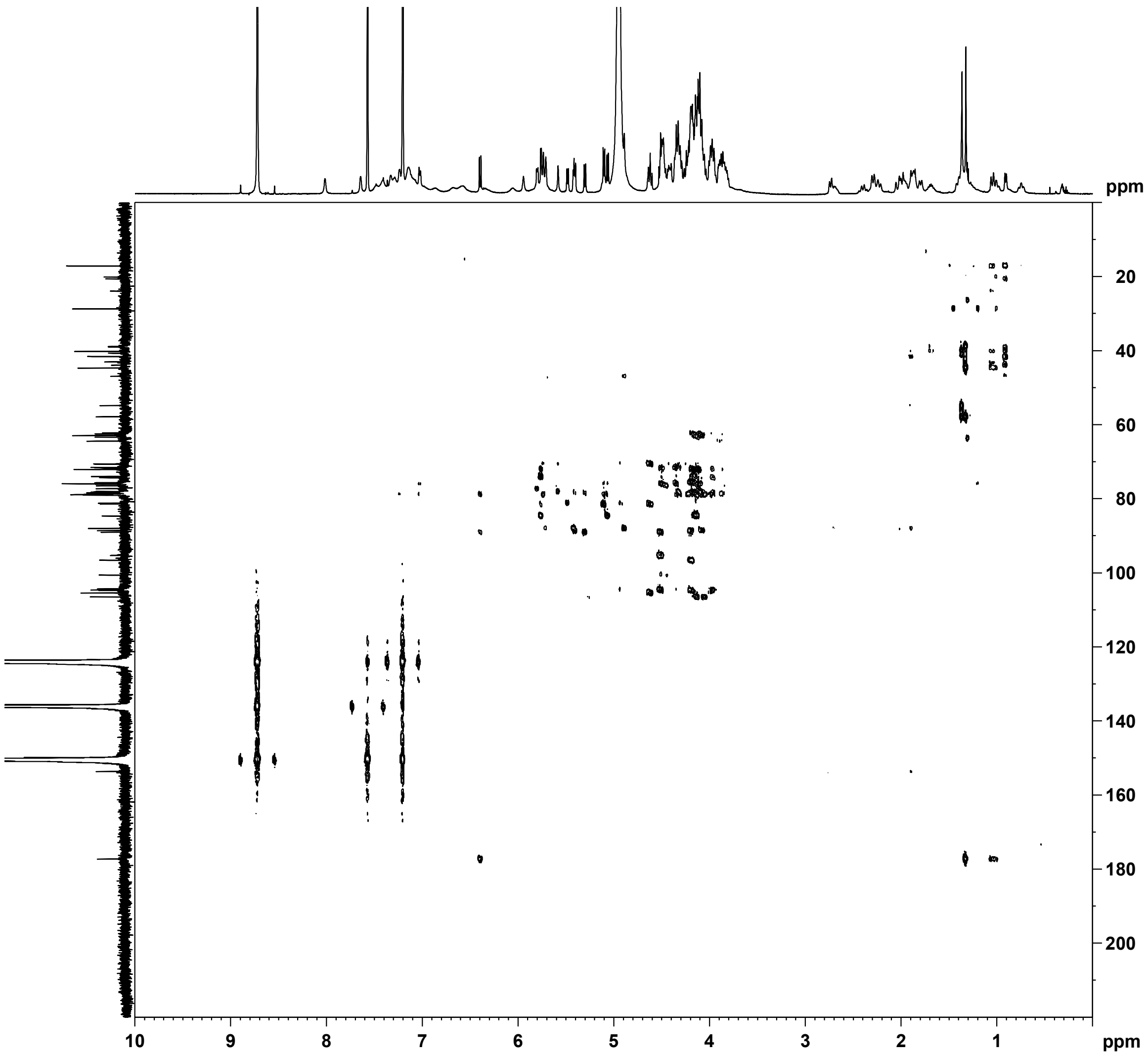
ppm
20
40
60
80
100
120
140
160
180
200
10
9
8
7
6
5
4
3
2
1
ppm

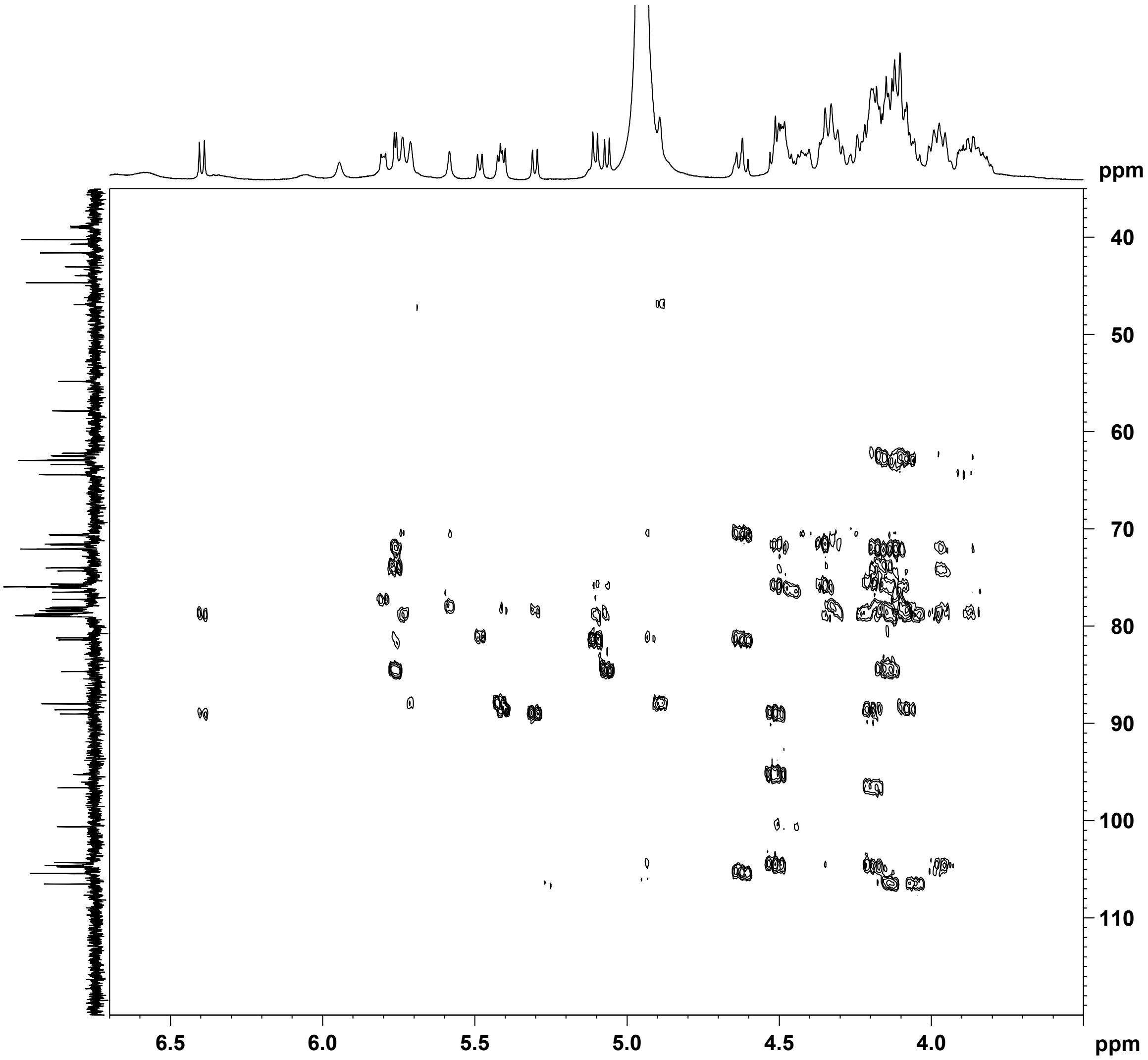

Figure S8. ^{1}H-[13]C Heteronuclear Multiple Bond Correlation (HMBC) spectra, full scale and expansion of Rebaudioside IX (**1**).

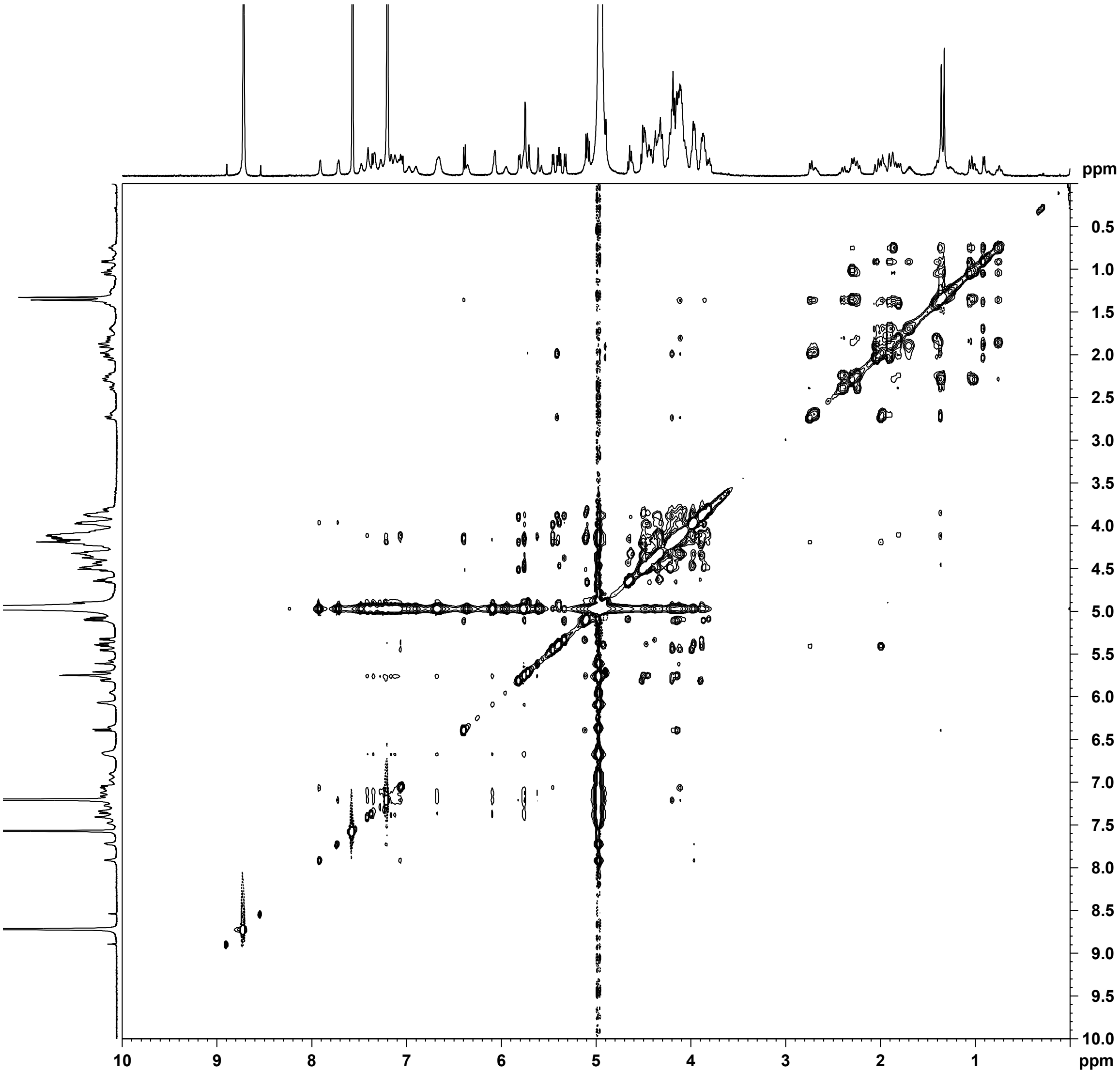

Figure S9. ^1H-^1H Nuclear Overhauser Effect Spectroscopy (NOESY) spectrum of Rebaudioside IX (**1**).
